# “Nailing” the diagnosis: Identification and treatment of subungual osteoma cutis with the use of point-of-care ultrasound

**DOI:** 10.1016/j.jdcr.2024.09.005

**Published:** 2024-09-23

**Authors:** Hira Asim, Pavela Bambekova, Brooke A. Burgess, Nilam Soni, Huma Siddiqui, Joshua L. Owen

**Affiliations:** a Long School of Medicine, University of Texas Health San Antonio, San Antonio, Texas; b Division of Dermatology, University of Texas Health San Antonio, San Antonio, Texas; c Medicine Service, South Texas Veterans Health Care System, San Antonio, Texas; d Pathology Service, South Texas Veterans Health Care System, San Antonio, Texas; e Dermatology Service, South Texas Veterans Health Care System, San Antonio, Texas

**Keywords:** cutaneous bony growth, exostosis, nail lesion, nail unit, osteoma cutis, periungual, point-of-care ultrasound, subungual, ultrasound in dermatology

## Introduction

The 2 pillars of the dermatologic examination are visual inspection and palpation. Various techniques have been added to augment the dermatologic examination, including biopsy, Wood lamp, and dermoscopy.^1^ Point-of-care ultrasound (POCUS), while recently employed in a wide variety of medical and surgical specialties, has had slower uptake and utilization in dermatology. POCUS is noninvasive and adds additional information to the clinical examination, including data on lesion character, consistency (echogenicity), vascularity, and depth.^1^ This report illustrates how use of POCUS assisted in a diagnostically challenging case of a painful subungual growth, initially thought to be an exostosis that proved to be osteoma cutis (OC).

## Case report

**Fig 1:**
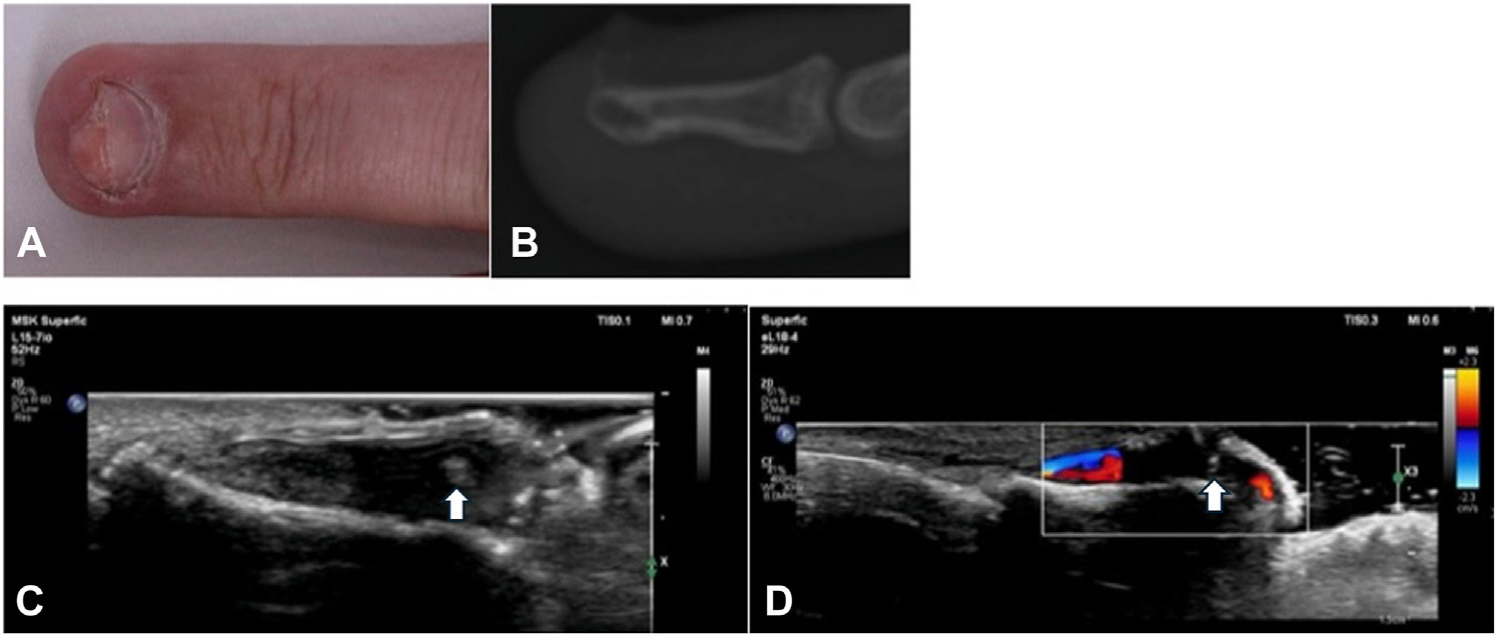
**A,** Initial clinical exam with a 6 mm skin-colored exophytic subungual papule on the distal tip of the right fourth finger without overlying epidermal changes. **B,** X-ray showed a 2 mm peripherally calcified low-density lesion overlying the dorsal tuft of the fourth digit distal phalanx. **C** and **D,** Ultrasound images of the right fourth digit using a high-frequency probe (*arrow* pointing to osteoma cutis). **C,** Nail bed appears hypoechoic with small round hyperechoic foci, consistent with calcification without any bony defect of the underlying distal phalanx. **D,** Doppler ultrasound demonstrating normal vascular flow without hypervascularity of the lesion.

A 41-year-old male presented with a 6-month history of a painful and enlarging papule on the right fourth finger. Exam revealed a 6 mm skin-colored, smooth, and tender subungual papule on the right fourth fingertip pushing upward on the central nail (Fig 1, *A*).

**Fig 2:**
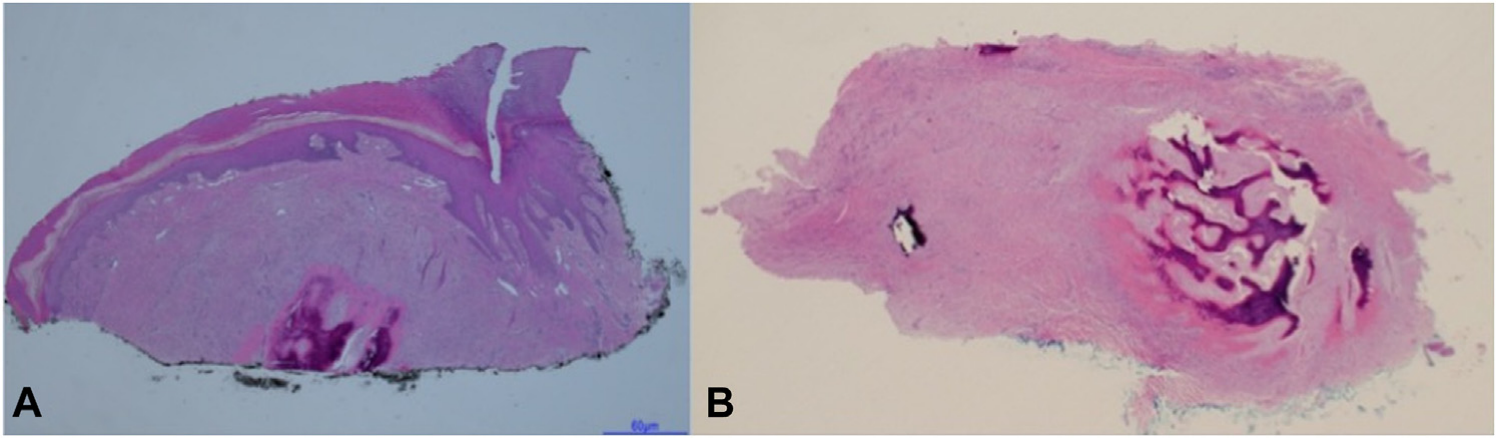
Histopathological images of subungual osteoma cutis. **A,** Shave biopsy of the initial lesion. Hematoxylin and eosin staining revealed a dome-shaped papule with deposits consistent with bony elements in the dermis (20×). **B,** Excisional specimen (hematoxylin and eosin, 40×). Bone formation is seen with a surrounding scar in the dermis.

The differential diagnosis included subungual exostosis but given the lesion’s nonclassical features (finger versus toe and central distal versus lateral nail location), other possible diagnoses including periungual or subungual fibroma, subungual verruca, squamous cell carcinoma, giant cell tumor of the tendon sheath, and schwannoma were considered. During a shave biopsy of the distal exophytic portion of the papule, a firm structure was encountered within the papule. Histopathology demonstrated bony elements within the dermis, consistent with subungual exostosis (Fig 2, *A*). As the bony elements were transected at the base of the biopsy specimen, a definitive diagnosis could not be made from the biopsy and an x-ray was performed (Fig 1, *B*). It revealed a 2 mm peripherally calcified low-density lesion overlying the dorsal tuft of the distal phalanx of the fourth digit, and the fourth digit distal phalanx appeared intact. These results were not consistent with subungual exostosis, and a calcified epidermal inclusion cyst was favored.

Given the conflicting data from the biopsy and radiography, the lesion was further evaluated by ultrasound using a high-frequency probe (Philips 5500 eL 18-4 probe). Ultrasonography revealed a hypoechoic area of nail bed with several small round hyperechoic foci, consistent with calcification (Fig 1, *C*). Cortex of the underlying distal phalanx appeared intact without any bony defects, and the lesion lacked hypervascularity by Doppler ultrasound (Fig 1, *D*). Putting together findings from biopsy, radiography, and ultrasonography, a diagnosis of OC was favored.

**Fig 3:**
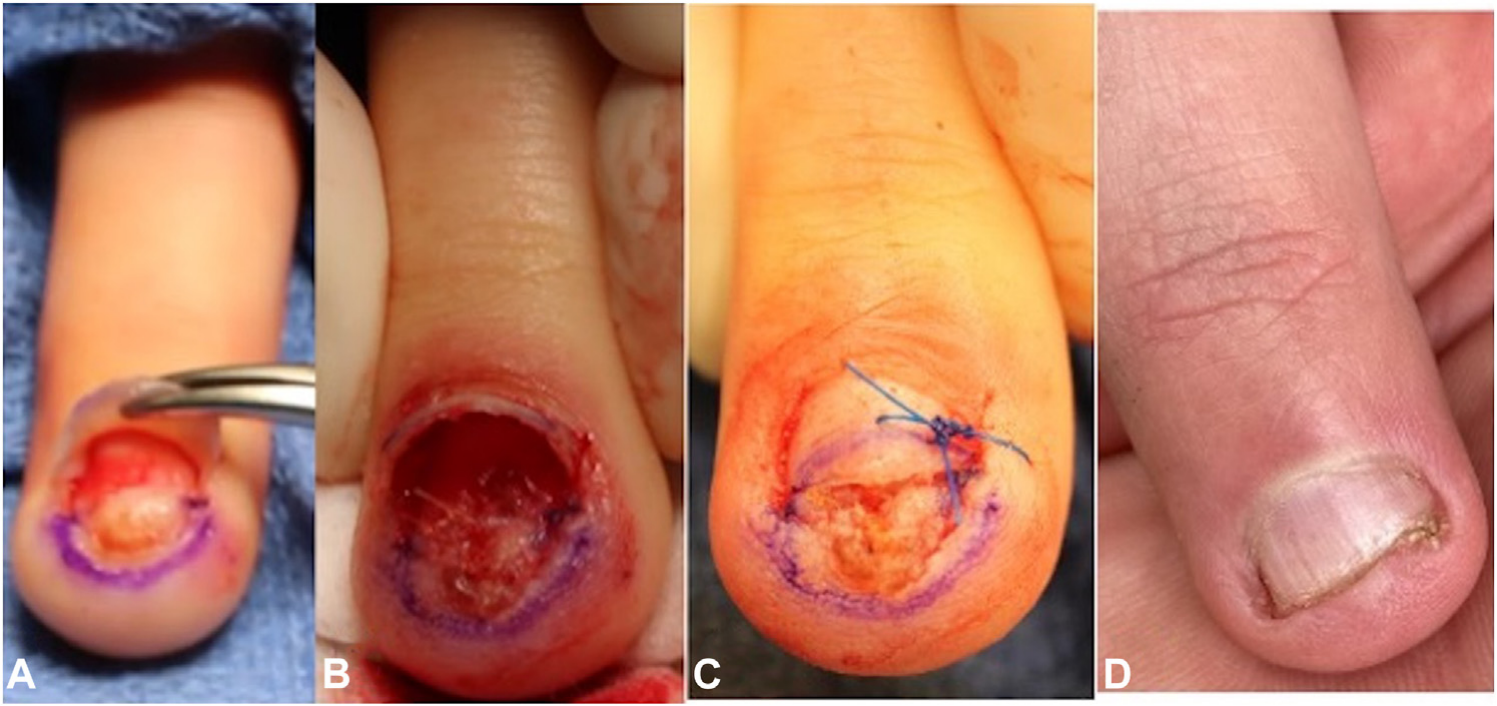
Clinical images. **A,** Clinical appearance of the lesion after trapdoor nail avulsion. **B,** Intraoperative image after lesion enucleation, excision, and primary nail bed repair. **C,** Immediate postoperative appearance with nail plate in situ. **D,** Follow-up 6 months postoperatively without recurrence or nail dystrophy.

As the lesion was tender, it was surgically excised (Video 1, available on www.jaad.org, Supplementary Material, available via Mendeley at https://data.mendeley.com/datasets/wkm679dxfk/1). After performing a distal digital block, trap door avulsion was performed followed by a longitudinal incision of the nail bed overlying the lesion (Fig 3, *A*). The lesion was easily dissected and enucleated from the overlying nail bed and underlying deep tissue/periosteum. Nail bed was repaired primarily, and nail plate was returned in situ (Fig 3, *B* and *C*). Histopathology confirmed subungual OC (Fig 2, *B*). At his 6-month follow-up appointment, the patient had healed well without postoperative complications, tenderness, recurrence, or nail dystrophy (Fig 3, *D*).

## Discussion

OC, also known as “cutaneous ossification,” refers to aberrant deposition of bone in the skin, which can occur after skin trauma, or less commonly, as a result of metabolic or inflammatory disease processes.^2^ OC differs from calcinosis cutis in that the former is derived from a membranous ossification process that does not involve cartilage as a precursor, while the latter is defined by the deposition of calcium salts without osteoid development.^3^ OC is a benign condition that may be classified as either primary or secondary. Primary OC arises in the absence of prior inflammatory or neoplastic changes in affected skin, while secondary OC is linked to chronic inflammatory states, trauma, and neoplasms.^3^ Our patient worked with hand tools and reported many injuries to his hands/fingers. A diagnosis of OC is generally confirmed by biopsy and histopathological evaluation, which reveals bone spicules in the dermis and subcutaneous tissue and microcalcifications.^4^ Prior to biopsy, high-frequency ultrasound can be used to help narrow the differential diagnosis and can aid in procedural planning, especially in locations with complex anatomy like the nail unit.

In the past decade, POCUS has been increasingly used in dermatology as an efficient, cost-effective imaging method to supplement visual and tactile elements of a traditional physical examination.^5^ Despite the versatility of this imaging modality in dermatology, POCUS still faces barriers to use. Perez-Sanchez et al reported that few dermatologists currently use POCUS due to lack of training, equipment, or infrastructure. Most important, lack of awareness of the broad range of clinical applications and potential benefits of dermatologic POCUS are critical initial barriers to use.^6^

Despite these barriers, dermatologic POCUS has been used in characterization and treatment of a variety of skin conditions (benign or malignant neoplasms, presurgical planning for skin cancers, evaluation and treatment of scars, staging of inflammatory disorders, cosmetics).^6^ Fewer cases have utilized POCUS for nail unit disorders, focusing primarily on investigation of glomus tumors, fibrous tumors, onychomatricomas, granulomas, subungual warts, mucous cysts, synovial cysts, and subungual exostoses.^7^

The nail unit has a relatively small diagnostic field and a complex anatomic structure, making POCUS and its submillimeter scale a useful method in analyzing subungual and periungual nail conditions.^5^ POCUS serves as a useful diagnostic tool compared to x-ray as it can better characterize lesion in terms of echogenicity (anechoic, hypoechoic, or hyperechoic), vascularity, internal characteristics (fluid-filled, cystic, solid), location, depth of involvement of nail unit components, and surrounding architecture. These features are then integrated with other elements of the clinical evaluation to formulate a treatment plan.^5^ In this case, ultrasonography provided additional information that x-ray did not, demonstrating a distinct calcified, nonvascular, and noncystic lesion, which aided in the final diagnosis of subungual OC and guided surgical planning.

Although POCUS cannot solely replace dermoscopy, histopathology, and radiography in the evaluation of nail unit lesions, POCUS can rapidly supplement other imaging and clinical findings to guide management. Given barriers identified to POCUS use in dermatology, development of training programs for both practicing dermatologists and dermatology residents is needed. Clinical use of dermatologic POCUS, as demonstrated by our case, can help “nail” certain dermatological diagnoses, including those of the nail unit.

## Conflicts of interest

None disclosed.

## Supplementary data

Video E1
